# Feasibility of Bladder Neck Incision in Female Patients With Primary Bladder Neck Obstruction: Our Experience

**DOI:** 10.7759/cureus.88771

**Published:** 2025-07-25

**Authors:** Saqib Mehdi, Abdul R Khawaja, Sajad A Malik, Sajad A Para, Arif Hamid

**Affiliations:** 1 Urology, Sher-i-Kashmir Institute of Medical Sciences, Srinagar, IND

**Keywords:** bladder neck incision, bladder outflow obstruction, primary bladder neck obstruction, urodynamic study, voiding dysfunction

## Abstract

Objectives

To study the feasibility of bladder neck incision (BNI) in female patients with primary bladder neck obstruction in whom conservative measures have failed to show promising results.

Materials and methods

We included 48 female patients who had all the following features at presentation: maximum flow rate of urine <12 mL/second, inadequate funneling of the bladder neck on voiding cysto-urethrogram, post-void residual urine (PVRU) >100 mL, sustained detrusor contraction of >25 cmH2O at maximum urinary flow rate (Qmax), and a normal urethral caliber. Uroflowmetry, voiding cystometrogram, and cystourethroscopy were performed preoperatively in all patients. The surgical procedure involved endoscopic incisions at five and seven o'clock positions on the bladder neck with bipolar diathermy using the TURis system. All patients underwent a postoperative symptom score assessment and uroflowmetry at the one-year follow-up.

Results

The mean age of the patients in our study was 47 years (range: 23 to 66 years), with a median follow up of 33.4 months. Preoperatively Qmax, mean International Consultation on Incontinence Questionnaire - Female Lower Urinary Tract Symptoms Long Form (ICIQ-FLUTS LF) score, PVRU, and mean quality of life score were 7.14 mL/sec, 19.4, 139 mL, and 4.5, respectively. Postoperatively, the same variables were 19.37 mL/sec, 7.6, 32 ml, and 2.3, respectively. Mild-to-moderate stress urinary incontinence was observed in four patients (<2 points).

Conclusion

In female patients, BNI offers a safe, effective, and minimally invasive treatment option. Our technique, when done as described, is easy to perform and has a minimal complication rate.

## Introduction

The bladder neck in women is described as a functional entity, which unlike men, lacks definite outer circular and inner longitudinal layers. This probably accounts for primary bladder neck obstruction (PBNO) being less common and under-reported in female patients [1]. The bladder neck acts in combination with the proximal urethra to maintain continence, while its failure to relax properly during voiding may constitute an important cause of female PBNO. The site of obstruction is confirmed by a closed bladder neck on fluoroscopy at the time of voiding. Although in recent years several nomograms, like the Solomon Greenwell nomogram, have been developed to better diagnose and validate bladder outlet obstruction (BOO) in female patients, they require the presence of a voiding cystogram, which may not be available at all centers. Therefore, many authors have described it as sustained high-pressure voiding with detrusor pressure at maximum urinary flow rate (PdetQmax) of 20-25 cmH2O in the presence of low maximum urinary flow rate (Qmax) of 11-15 mL/sec [2,3].

In female patients with BOO, literature mentions an increasing use of alpha blockers [4], which leads to an improvement in more than 50% of the patients [5]. For patients who fail to improve on alpha blockers, conservative management relies mainly on clean intermittent self-catheterization (CISC) to cause bladder emptying and maintain low bladder pressures. Surgical intervention in the form of bladder neck incision (BNI), as in males, may therefore offer a permanent solution. The procedure essentially involves division of the bladder neck at various positions to mechanically relieve the outlet obstruction. We present our experience of BNI in 48 female patients, along with their follow-up and complications.

## Materials and methods

This is a retrospective study carried out at our institute (Sher-i-Kashmir Institute of Medical Sciences (SKIMS), Srinagar, Kashmir, India), which is a tertiary care referral center in our region. The study was carried out by reviewing the hospital records of the patients who fulfilled the inclusion criteria over the past six years.

**Figure 1:**
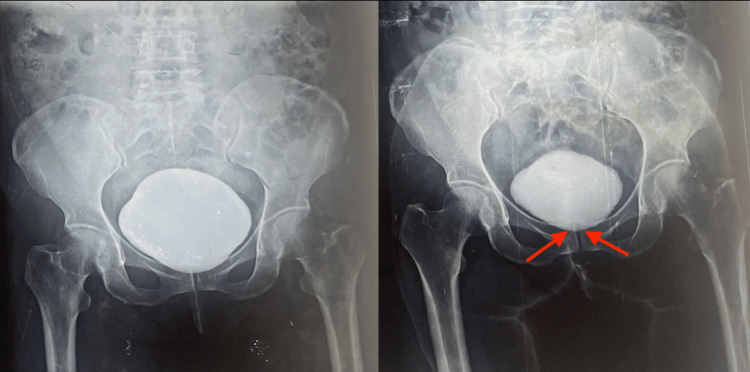
Voiding cysto-urethrogram showing inability of the bladder neck (marked with arrows) to open adequately on voiding

Most of the patients had initially presented with frequency, hesitancy, and straining at micturition, for which they were being treated with alpha blockers. They had responded poorly to the medication, and some of them had been put on CISC, which was met with poor compliance. Due to the poor results following conservative measures, shared decision-making between the patient and the treating urologist led to the decision for a surgical procedure. We included female patients who had all the following features at presentation: maximum flow rate of urine <12 mL/second, inadequate funneling of the bladder neck on voiding cysto-urethrogram (Figure 1), post-void residual urine (PVRU) >100 mL, sustained PdetQmax >25 cmH2O and a normal urethral caliber.

The absence of urethral stricture disease/normal caliber of the urethra were confirmed with gentle urethral calibration. All urodynamic studies were performed while complying with the International Continence Society (ICS) good urodynamic practice recommendations [6]. Patients with a neurogenic bladder and history of trauma were excluded.

A comprehensive evaluation of each patient was carried out, which comprised of complete history including an International Consultation on Incontinence Questionnaire - Female Lower Urinary Tract Symptoms Long Form (ICIQ FLUTS LF) [7], physical examination, and a focused neurological examination. Local examination to rule out anatomic causes of obstruction like prolapse, any mass lesion or meatal stenosis was also carried out. Baseline blood investigations, urine examination, urine culture, uroflowmetry, voiding cystometrogram, and cystourethroscopy were performed in all patients before surgery. A single dose of a third-generation intravenous antibiotic was given before surgery.

The surgical procedure involved endoscopic incisions at five and seven o'clock positions on the bladder neck with bipolar diathermy using TURis system (Olympus, Tokyo, Japan), with a Collins knife through 24 French resectoscope. The incision was started just inside the urinary bladder at the bladder neck staying quite away from the ureteric orifice. It was continued through the proximal third of urethra and stopped just before reaching mid urethra. At the time of cystoscopy, done before the procedure, an approximate length of urethra was kept in mind. This prevented us from overshooting while making endoscopic incisions in the absence of a definite anatomic landmark like the verumontanum. Once the initial incision was made, we checked whether the incision was of adequate length or there was a need to increase its length. Both incisions were deepened till we were convinced of dividing the circular fibres, but did not deepen our incisions till the perivesical fat became visible. Point coagulation of all the active bleeders was achieved intra-operatively. An 18 Fr Foley catheter was placed post procedure for a period of three days in all patients, who were discharged on the first post-operative day. Pain was managed with intravenous paracetamol on the day of procedure, while patients were advised to take oral paracetamol in case of pain after discharge from hospital. All patients were subjected to a postoperative symptom score and uroflowmetry during follow-up at three and twelve months. All the preoperative and postoperative variables, including complications, were documented.

The available data was analyzed statistically with IBM SPSS Statistics for Windows, Version 26 (Released 2019; IBM Corp., Armonk, New York, United States). Preoperative and postoperative variables like Qmax, PVRU, ICIQ FLUTS LF score, and quality of life (QOL) were compared using paired t-test. The differences were considered statistically significant only at a P value of less than 0.05.

## Results

This study included 48 female patients studied over a period of six years who fulfilled the inclusion criteria. They were aged between 23 to 66 years with a mean age of 47 years. The patients had a mean body mass index of 27.3, three patients were hypertensive, three were hypothyroid, and one was diabetic. Forty five patients (87.5%) presented with complaints of frequency, hesitancy, and straining at micturition. Recurrent urinary tract infections (UTIs) were seen in 11 patients (22.9%) and, vesico-ureteral reflux on voiding cystourethrogram was seen bilaterally in two patients (4%) and unilaterally in four patients (8%). Preoperatively, all the patients had normal serum creatinine. Alpha blockers were discontinued at least two weeks prior to surgery in patients who were continuing on them. Fourteen patients had been on CISC preoperatively (29.2%). None of our patients had a history of acute urinary retention, stress, or urge urinary incontinence. All the patients were followed up for a minimum period of one year. The median follow up of patients in the study was 33.4 months. On comparing the preoperative and postoperative variables recorded at 12 months a statistically significant difference was noted in Qmax, ICIQ FLUTS LF score, mean PVRU, and mean QOL score (Table 1). 

**Table 1:** Preoperative and postoperative variables PVRU: post-void residual urine; ICIQ FLUTS LF: International Consultation on Incontinence Questionnaire - Female Lower Urinary Tract Symptoms Long Form; QOL: quality of life

Variable	Preoperative	Postoperative	P value
Qmax (mL/second)	7.14	19.37	<0.01
PVRU (mL)	139	32	<0.01
ICIQ FLUTS LF	19.4	7.6	<0.01
QOL	4.5	2.3	<0.01

The procedure was successful in the first attempt in 87.5% of the patients. Six patients who had a Qmax of <10 mL/sec postoperatively, with recurrent/persistent symptoms underwent a repeat procedure. An improvement in the voiding function and Qmax was noted in these patients after the repeat procedure. Although all the patients enrolled in the study experienced an improvement in their voiding symptoms, the Qmax in six patients with vesicoureteric reflux improved to a mean of only 11.53 mL/sec as compared to an overall mean of 19.37 mL/sec. The 11 patients (22.9%) who had experienced UTIs preoperatively did not report any such episodes in the postoperative period.

Stress urinary incontinence (SUI) was diagnosed clinically in four patients (8%) postoperatively. All these patients had mild or moderate SUI as per ICIQ FLUTS LF questionnaire score standard (one or two points) and were managed conservatively with duloxetine without the need for any surgical intervention. The age of these patients ranged from 43 to 62 years with a mean of 53 years.

None of the patients in our study required blood transfusions and post-operative hematuria, if any, resolved with saline irrigation. Post-procedural fever or infection was not seen in any of our patients. None of the patients in our study developed urethral stricture or vesiovaginal fistula following surgery.

## Discussion

While the first BNI was described about five decades ago by Turner-Warwick et al. [8], its use has been more widespread in male patients as compared to female ones. Although it is primarily due to lower rates of bladder outlet obstruction in females owing to anatomic differences between the two genders, many cases of female BOO go unnoticed due to lack of a proper diagnosis of BOO [9-11]. While the diagnosis of PBNO in females is usually based on a video urodynamic study, its unavailability at many health facilities highlighted the need for basing our diagnosis on other parameters. We based it on sustained high voiding pressure in the presence of low urine flow rate, complemented by a closed bladder neck on voiding cystourethrogram. We excluded all other causes which could contribute to obstructed voiding in the patients. Men and women have different characteristics of micturition. What may be a normal flow rate and voiding pressure for women may not always qualify as normal for men. The nomograms for diagnosing BOO in men have been developed based on clinical presentation and response to treatment of men with benign prostatic obstruction (BPO). Female patients do not present with common conditions like BPO and BPH. Therefore, definitions and nomograms devised to describe BOO in male patients are not applicable in female patients. The Solomon Greenwell nomogram based on video urodynamics has been used to describe the probability of BOO in female patients [3]. The nomogram plots Pdet@Qmax against Qmax, and an axis identified as Pdet@Qmax = 2Qmax is used to separate obstructed from unobstructed cases. In our study, on retrospectively plotting the cases on the Solomon Greenwell nomogram, 44 cases were found to lie above the axis. Thus, 90% of the cases in our study were found to be obstructed as per the Solomon Greenwell nomogram.

We included only those patients in our study in whom surgical therapy was deemed necessary due to failure to respond to pharmacotherapy. The British Association of Urological Surgeons (BAUS) panel has not recommended BNI as treatment for female BOO in the BAUS consensus document on management of female voiding dysfunction. It has, however, been described as a treatment option which can be offered in selected patients at subspecialist tertiary care centers [12]. We don’t consider this statement to be completely against the parameters used in our study. Our hospital is a tertiary care referral hospital which has been practicing female urology for more than a decade. Patients were included in the study only after excluding other causes of BOO and after they failed to respond to conservative measures. Moreover, literature includes several prospective case series that have all shown significant improvements in symptom scores, quality of life, Qmax, and PVRU after BNI compared to baseline [13-16].

There are many factors that are hypothesized to lead to obstruction at the level of the bladder neck. Hypertrophy/fibrosis of the bladder neck and dysynergia between the bladder neck and detrusor muscles have been attributed to be important causes; the exact predisposing factor still remains unclear [17]. While BNI essentially involves division of this ring-like structure formed by the bladder neck, the authors seem to be divided about the location of incisions. Turner-Warwick et al. used to make a single incision at the 12 o'clock position for the fear of creating vesicovaginal fistulas with posterior or posterolateral incisions [8]. Such incisions run the risk of incomplete resolution of obstruction, while the concerns for fistulation were unfounded in our study. We made incisions at five and seven o'clock positions in all our patients, but none of them developed a vesicovaginal fistula. Similar findings were noted by Blaivas et al. who did not experience vesicovaginal fistulas in any of their patients following bladder neck incisions at five and seven o'clock positions [18]. To circumvent the risk of fistulation, Jin et al. [16] described a modified BNI followed by regular urethral dilatations. They made incisions at three, six, nine and 12 o'clock positions. We consider this procedure technically more difficult for the surgeon and tedious for the patient as she has to continue with regular urethral dilatations. Ours is a relatively simple procedure as most urologists are quite used to making similar incisions in male BNI. We extended the length of the incisions up to mid urethra and depth was not extended just after beginning to see the fat. We experienced a very good success rate of 87.5% with this procedure with only six patients requiring a second BNI. The success rate in our study was comparable to that of other major studies in the literature [13,15,18].

We did a repeat BNI in patients who had a Qmax <10ml/sec with recurrent/persistent symptoms. Most of the complications were minor and self limiting. None of the patients in our study required blood transfusions, and post-operative hematuria, if any, resolved with saline irrigation. While Zhang et al. [13] required urethral dilatations in three patients in the postoperative period, we did not encounter any such case. This is probably explained by the fact that we carefully excluded stricture disease preoperatively in all the patients before surgery. We encountered two patients who had co-existent urethral stricture with a high bladder neck. They underwent a buccal mucosal graft urethroplasty (BMGU) followed by BNI, but were not included in the current study. SUI seen in four of our patients resolved with pharmacotherapy (duloxetine), with none of the patients requiring a procedure such as synthetic mid urethral sling for it. Thus, the need to do a repeat surgery can be considered to be the most common major complication in our set of patients. We tried to err on the side of doing a repeat procedure than rendering the patient incontinent, as treating incontinence after the procedure required a more challenging procedure than doing a repeat BNI. While compiling the results of our study and comparing it with other studies in literature we strongly feel that the rate of incontinence in female patients post BNI is linked more with depth and length of incisions than their location. Even when incisions are made posteriorly but not extended deeper into the fat and distal urethra, the patient can be relieved of obstruction without rendering her incontinent.

The current study has certain limitations. It is retrospective in nature and a single arm study. Further studies enrolling greater number of patients, and comparing them with other techniques will be required in future to establish the effectiveness of our technique in the long term vis-à-vis other techniques.

## Conclusions

PBNO is an important cause of lower urinary tract symptoms in female patients. The diagnosis can be confirmed on the basis of a voiding cysto-urethrogram complemented by a urodynamic study, after excluding other causes of BOO. BNI can be offered to these patients when they have failed conservative treatment options.

When done as described, BNI offers a safe and effective treatment option, yielding very good results in the majority of these patients. Our technique is very easy to perform as urologists are already well versed with it, and has a minimal complication rate without any major morbidity.
